# Succession of *Bifidobacterium longum* Strains in Response to a Changing Early Life Nutritional Environment Reveals Dietary Substrate Adaptations

**DOI:** 10.1016/j.isci.2020.101368

**Published:** 2020-07-15

**Authors:** Magdalena Kujawska, Sabina Leanti La Rosa, Laure C. Roger, Phillip B. Pope, Lesley Hoyles, Anne L. McCartney, Lindsay J. Hall

**Affiliations:** 1 Gut Microbes & Health, Quadram Institute Biosciences, Norwich Research Park, Norwich NR4 7UQ, UK; 2 Faculty of Chemistry, Biotechnology and Food Science, Norwegian University of Life Sciences, 1433 Aas, Norway; 3 Faculty of Biosciences, Norwegian University of Life Sciences, 1433 Aas, Norway; 4 Department of Biosciences, Nottingham Trent University, Nottingham NG11 8NS, UK; 5 Department of Food & Nutritional Sciences, University of Reading, Reading RG6 6LA, UK; 6 Norwich Medical School, University of East Anglia, Norwich Research Park, Norwich NR4 7TJ, UK; 7 Chair of Intestinal Microbiome, School of Life Sciences, Technical University of Munich, 85354 Freising, Germany; 8 ZIEL – Institute for Food & Health, Technical University of Munich, 85354 Freising, Germany

**Keywords:** Dietary Supplement, Microbiology, Microbiome

## Abstract

Diet-microbe interactions play a crucial role in modulation of the early life microbiota and infant health. *Bifidobacterium* dominates the breast-fed infant gut and may persist in individuals during transition from a milk-based to a more diversified diet. Here, we investigated adaptation of *Bifidobacterium longum* to the changing nutritional environment. Genomic characterization of 75 strains isolated from nine either exclusively breast- or formula-fed (pre-weaning) infants in their first 18 months revealed subspecies- and strain-specific intra-individual genomic diversity with respect to carbohydrate metabolism, which corresponded to different dietary stages. Complementary phenotypic studies indicated strain-specific differences in utilization of human milk oligosaccharides and plant carbohydrates, whereas proteomic profiling identified gene clusters involved in metabolism of selected carbohydrates. Our results indicate a strong link between infant diet and *B*. *longum* diversity and provide additional insights into possible competitive advantage mechanisms of this *Bifidobacterium* species and its persistence in a single host.

## Highlights

• *B. longum* strains can persist in individual infants despite dietary changes
• New strains with additional metabolism capabilities can also be acquired
• *B. longum* carbohydrate metabolism repertoires are individual and strain-specific
• Strains at different dietary stages show genomic adaptations to specific substrates

## Graphical Abstract

**Figure:**
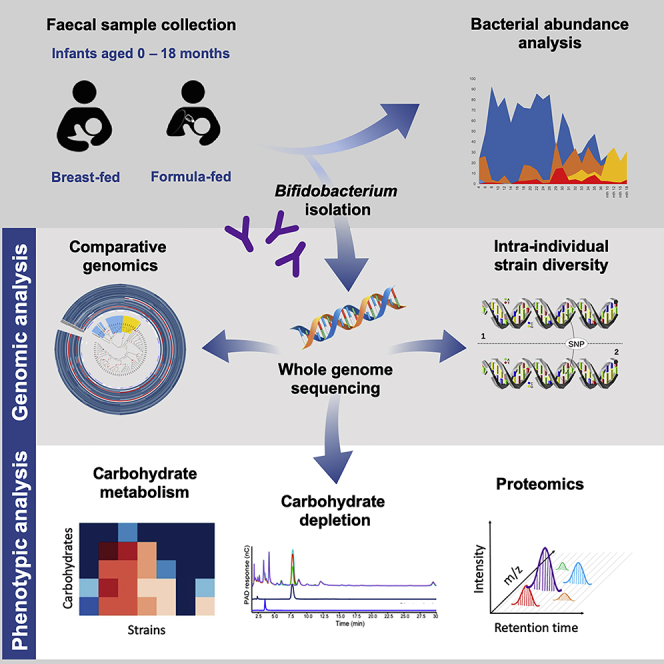

## Introduction

Microbial colonization shortly after birth is the first step in establishment of the mutualistic relationship between the host and its microbiota (Backhed et al., 2015; Wampach et al., 2017; Lawson et al., 2020). The microbiota plays a central role in infant development by modulating immune responses, providing resistance to pathogens, and also digesting the early life diet (Heikkila and Saris, 2003; Sela et al., 2008; Marcobal and Sonnenburg, 2012; Sivan et al., 2015; de Aguero et al., 2016; Thongaram et al., 2017). Indeed, diet-microbe interactions are proposed to play a crucial role during infancy and exert health effects that extend to later life stages (Turnbaugh et al., 2006; Renz et al., 2012; Olszak et al., 2012; Feng et al., 2015; Bokulich et al., 2016; Tang et al., 2017). The gastrointestinal tract of vaginally delivered full-term healthy infants harbors a relatively simple microbiota characterized by the dominance of the genus *Bifidobacterium* (Dogra et al., 2015; Shao et al., 2019). In contrast, caesarean-section-born infants have disrupted transmission of maternal gastrointestinal bacteria, such as *Bifidobacterium*, and high levels of opportunistic hospital-associated pathogens (Shao et al., 2019).

Breast milk is considered the gold nutritional standard for infants, which also acts as an important dietary supplement for early life microbial communities, including *Bifidobacterium*. The strong diet-microbe association has further been supported by reports of differences in microbial composition between breast- and formula-fed infants (e.g. high versus low *Bifidobacterium* abundance) and related differential health outcomes between the two groups: e.g. increased instances of asthma, allergy, and obesity in formula-fed infants (Ip et al., 2007; Das, 2007; O'sullivan et al., 2015; Martin et al., 2016; Stiemsma and Michels, 2018; Ortega-Garcia et al., 2018; Forbes et al., 2018).

The high abundance of *Bifidobacterium* in breast-fed infants has been linked to the presence of specific carbohydrate utilization genes and gene clusters in their genomes, particularly the ones involved in the degradation of breast milk-associated human milk oligosaccharides (HMOs) (Sela et al., 2008). The presence of these genes is often species- and indeed strain-specific and has been described in *Bifidobacterium breve*, *Bifidobacterium bifidum*, *Bifidobacterium longum*, *Bifidobacterium infantis*, and more rarely in *Bifidobacterium pseudocatenulatum* (Sela et al., 2008; James et al., 2016; Katayama, 2016; Garrido et al., 2016). However, previous studies have indicated co-existence of *Bifidobacterium* species and strains in individual hosts, resulting in interaction and metabolic co-operation within a single (HMO-associated) ecosystem (Milani et al., 2015a; Lawson et al., 2020).

Transition from breastfeeding to a more diversified diet and the introduction of solid foods has been considered to initiate the development of a functionally more complex adult-like microbiome, including presence of genes responsible for degradation of plant-derived complex carbohydrates, starches, and xenobiotics, as well as production of vitamins (Koenig et al., 2011, Mckeen et al., 2019). Non-digestible complex carbohydrates such as inulin-type fructans (ITF), arabinoxylans (AX), or arabinoxylo-oligosaccharides (AXOS) in complementary foods have been proposed to potentially exert beneficial health effects through their bifidogenic and prebiotic properties and resulting modulation of the intestinal microbiota and metabolic end-products (Roberfroid, 2007; Broekaert et al., 2011; Hald et al., 2016; Riviere et al., 2016).

Despite the shift in microbiota composition during weaning, specific strains of *Bifidobacterium*, and *B*. *longum* in particular, have previously been shown to persist in individuals over time (Maldonado-Gomez et al., 2016; Oki et al., 2018). *B*. *longum* is currently recognized as four subspecies: *longum* and *infantis* (characteristic of the human gut microbiota) and *suis* and *suillum* (from animal hosts) (Mattarelli et al., 2008; Yanokura et al., 2015). It is considered the most common and prevalent species found in the human gut, with *B*. *longum* subsp. *infantis* detected in infants, and *B*. *longum* subsp. *longum* widely distributed in both infants and adults (Turroni et al., 2009, 2012). The differences in prevalence between the two subspecies and the ability of infant, adult, and elderly hosts to acquire new *B*. *longum* strains during a lifetime have been attributed to distinct bacterial carbohydrate utilization capabilities and the overall composition of the resident microbiota (Garrido et al., 2012; Odamaki et al., 2018).

There have been several recent studies that have explored the early life microbiota in breast- and formula-fed babies (Magne et al., 2006; Palmer et al., 2007; Roger and Mccartney, 2010; Roger et al., 2010). Strain-level metagenomic investigation of the DIABIMMUNE cohort provided insights into diet-related functional aspects of *B*. *infantis* in breast-fed infants (Vatanen et al., 2019). Longitudinal studies focusing specifically on *B*. *longum* have highlighted intraspecies diversity, colonization, and long-term persistence (years) of this species in hosts; however, there have been limited investigations into diet-related functions at early life stages (Chaplin et al., 2015; Oki et al., 2018; Odamaki et al., 2018). Furthermore, although there are studies examining *B*. *longum* strains in relation to diet, these have not been profiled over longitudinal and changing dietary periods (Arboleya et al., 2018). Hence, longitudinal assessments of *B*. *longum* strains in single hosts over time, with focus on changing dietary patterns, are lacking, and further detailed studies are required.

Here, we investigate the adaptations of *Bifidobacterium* to the changing infant diet and examine a unique collection of *B*. *longum* strains isolated from nine infants across their first 18 months, encompassing pre-weaning, weaning, and post-weaning phases. We probed the genomic and phenotypic similarities between 62 *B*. *longum* strains and 13 *B*. *infantis* strains isolated from either exclusively breast-fed or formula-fed infants (pre-weaning). Our results indicate a strong link between host diet and *Bifidobacterium* species/strains, which appears to correspond to the changing nutritional environment.

## Results

Previous investigations into *B*. *longum* across the human lifespan have determined a broad distribution of this species, including prolonged periods of colonization (Maldonado-Gomez et al., 2016; Oki et al., 2018). To gain insight into potential mechanisms facilitating these properties during the early life window, we investigated the genotypic and phenotypic characteristics of *B*. *longum* strains within individual infant hosts in relation to diet (i.e. breast milk versus formula) and dietary stages (i.e. pre-weaning, weaning and post-weaning), following up on a longitudinal study of the infant fecal microbiota published in 2010 (Roger and Mccartney, 2010). Briefly, fecal samples from exclusively breast-fed infants and exclusively formula-fed infants were collected regularly from 1 month to 18 months of age (Roger and Mccartney, 2010). The number of samples obtained from the breast-fed infants during the pre-weaning period was higher than that obtained from the formula-fed group, which may correlate with differences in weaning age (~20.6 versus ~17 weeks old). Collected samples were subjected to quantitative analysis using fluorescence *in situ* hybridization (FISH) to enumerate the predominant bacterial groups (Table S1) (Roger and Mccartney, 2010). Bacterial isolation was also carried out on selected samples and the isolated colonies identified using ribosomal intergenic spacer analysis (Roger and Mccartney, 2010).

### Quantitative Analysis of Microbial Communities in Breast- and Formula-Fed Infants

**Figure 1:**
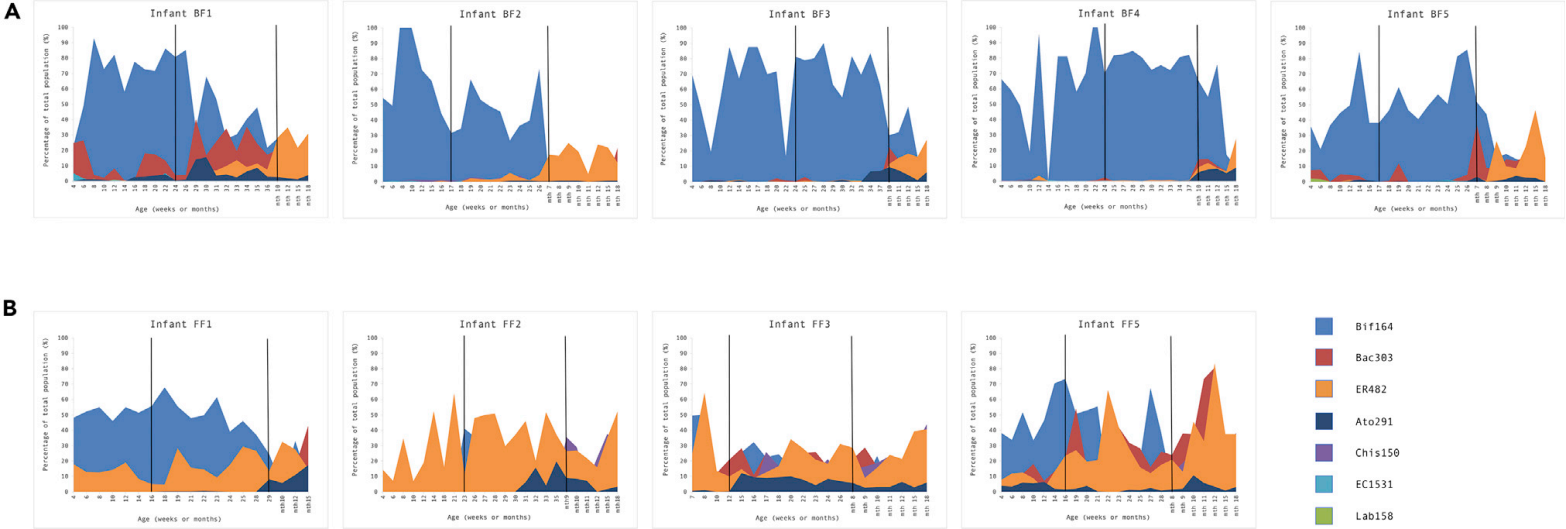
Proportional Representation of Bacterial Populations in the Fecal Microbiota of Infants Based on FISH analysis in (A) breast-fed and (B) formula-fed infants. Numbers are expressed as percentage of the total bacterial population obtained using DAPI. The vertical solid black lines mark the different dietary phases in each infant (pre-weaning, weaning, and post-weaning). Oligonucleotide probes used to determine bacterial populations: Bif164—most *Bifidobacterium* species and *Parascardovia denticolens*; Bac303—most members of the genus *Bacteroides*, some *Parabacteroides* and *Prevotella* species, *Paraprevotella*, *Xylanibacter*, *Barnesiella* species, and *Odoribacter splanchnicus*; ER482—most members of Clostridium cluster XIVa; Ato291—*Cryptobacterium curtum*, *Gordonibacter pamelaeae*, *Paraeggerthella hongkongensis*, all *Eggerthella*, *Collinsella*, *Olsenella* and *Atopobium* species; Chis150—most members of *Clostridium* cluster I, all members of *Clostridium* cluster II; EC1531—*Escherichia coli*; Lab158—all *Oenococcus*, *Vagococcus*, *Melissococcus*, *Tetragenococcus*, *Enterococcus*, *Catellicoccus*, *Paralactobacillus*, *Pediococcus* and *Lactococcus* species, most *Lactobacillus*, *Weissella*, and *Leuconostoc* species. See also Table S1.

To provide context to the microbiome environment the strains selected for the present study, we reanalyzed the data originally generated by FISH (Figure 1 and Table S1) (Roger and Mccartney, 2010). Bacteria detected using probe Bif164 (bifidobacteria) proportionally constituted the predominant group in samples isolated from breast-fed infants during pre-weaning and weaning: between 16.5% and 100% of the microbiota across the study period. During post-weaning, proportions of bifidobacteria across all breast-fed samples decreased considerably and ranged from 4.6% to 12.1%. The levels of bacteria detected by ER482 (members of *Clostridium* cluster XIVa) started to increase during weaning, increasing to 18.2% (from 0.25% at pre-weaning). Bacteria detectable by probe Bac303 (members of genus *Bacteroides*, *Parabacteroides* and *Prevotella* species, *Paraprevotella*, *Xylanibacter*, *Barnesiella* species and *Odoribacter splanchnicus*) were identified in all samples throughout the study, with this bacterial group showing extensive inter-individual variation. Other microbiota members were detected in breast-fed samples at lower levels, including members of family Coriobacteriia (Ato291, mean < 2% of microbiota), *Escherichia coli* (EC1531, <1%), members of *Clostridium* clusters I and II (Chis150, <1%), and lactic acid bacteria (Lab158, mean < 1%).

In contrast to the breast-fed group, no drastic shift in bacterial populations was observed in formula-fed infants throughout the study. Overall, lower levels of bifidobacteria were detected during pre-weaning and weaning, fluctuating from 0.0% to 73.3% of the microbiota at different time points. Similar to the breast-fed group, proportions of *Bifidobacterium* decreased during post-weaning across all formula-fed samples and ranged from 6.5% to 12% at month 18. The levels of bacteria detected by probe ER482 were overall higher in formula-fed samples throughout study duration: 19.96 ± 17.41%, 25.39 ± 14.63%, and 30.6 ± 15.92% for pre-weaning, weaning, and post-weaning phases. Similarly, proportions of bacteria detected by Bac303 during all dietary phases were higher in the formula-fed group compared with the breast-fed group. Contrastingly to the breast-fed group, levels of bacteria detected by Chis150 (*Clostridium* clusters I and II) started to increase during weaning in the formula-fed group and continued to increase (1.23 ± 1.28%, 7.03 ± 9.18%, and 21.72 ± 11.47% for pre-weaning, weaning, and post-weaning, respectively). Levels of bacteria identified by Ato291 and EC1531 in formula-fed samples were slightly higher than in the breast-fed group (means of <3.5% and <1.25%, respectively), whereas the mean proportion of lactic acid bacteria (Lab158) remained below 1%.

Overall, these results confirm previous studies that have indicated differences in fecal microbiota composition between breast- and formula-fed babies, particularly during the pre-weaning and weaning phases, and demonstrate the succession of bacterial species over time and in relation to diet, including *Bifidobacterium*.

### General Features of *B*. *longum* Genomes

**Figure 2:**
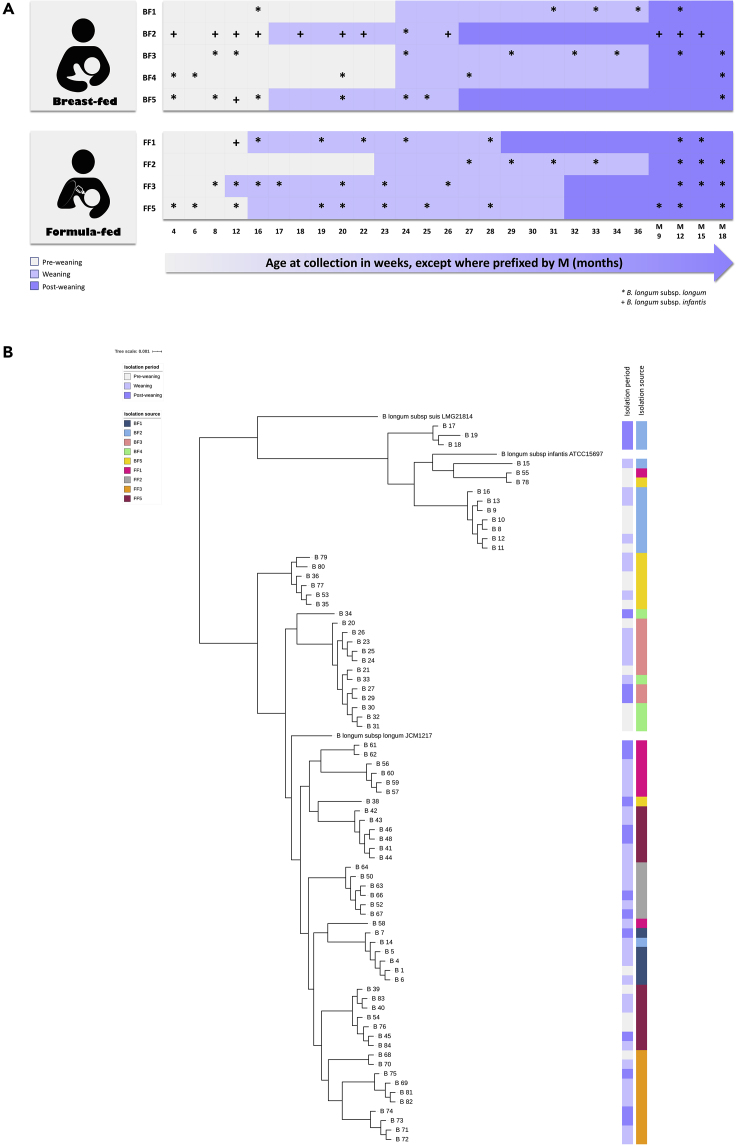
Identification and Relatedness of *B*. *longum* Strains (A) Sampling scheme and strain identification within individual breast-fed (BF1-BF5) and formula-fed (FF1-FF3 and FF5) infants based on average nucleotide identity values (ANI). The three levels of shading mark different dietary phases: pre-weaning, weaning, and post-weaning. (B) Relatedness of *B*. *longum* strains based on core proteins. Colored strips represent isolation period (pre-weaning, weaning, and post-weaning) and isolation source (individual infants), respectively. See also Tables S2, Table S3, and S4.

Based on the results of bacterial culture and colony identification published previously (for details, refer to (Roger et al., 2010)), 88 isolates originally identified as *Bifidobacterium* were selected for this study, 46 from five exclusively breast-fed infants (BF1-BF5, including identical twins BF3 and BF4) and 42 from four exclusively formula-fed infants (FF1-FF3 and FF5). Following sequencing and ANI analysis (Tables S2 and S3), 75 strains were identified as *B*. *longum* sp. and included in further analysis, with 62 strains identified as *B*. *longum* subsp. *longum* (*B*. *longum*) and 13 strains identified as *B*. *longum* subsp. *infantis* (*B*. *infantis*) (Figure 2A).

To determine possible genotypic factors facilitating establishment and persistence of *B*. *longum* in the changing early life environment, we assessed the genome diversity of our strains. Sequencing generated between 12 and 193 contigs for each *B*. *longum* strain, with 74/75 containing fewer than 70 contigs, yielding a mean of 66.95-fold coverage for strains (Table S2). The predicted genome size for strains identified as *B*. *longum* ranged from 2.21 Mb to 2.58 Mb, possessing an average G + C% content of 60.11%, an average predicted ORF number of 2,023, and number of tRNA genes ranging from 55–88. For strains identified as *B*. *infantis*, the predicted genome size ranged from 2.51 Mb to 2.75 Mb, with an average G + C% content of 59.69%, an average predicted ORF number of 2,280, and the number of tRNA genes ranging from 57 to 62.

### Comparative Genomics

To identify *B*. *longum* strains among the sequenced isolates and assess nucleotide-level genomic differences, we performed ANI analysis. Results (Table S3) indicated that *B*. *longum* strains isolated from individual infant hosts displayed higher levels of sequence identity than strains isolated from different hosts. More specifically, pairwise identity values for strains isolated from infant BF3 showed the narrowest range (average value of 99.99 ± 3.15 × 10^−5^%), followed by infant FF2 strains (99.98 ± 1.12 × 10^−4^%), with infant BF2 strains having the broadest identity value range (averaging 99.13 ± 7.8 × 10^−3^%).

Next, we examined genetic diversity of newly sequenced *B*. *longum* strains and their relatedness to each other, alongside *B*. *longum* type strains. We identified a total of 1,002 core genes present in at least 99% of the analyzed *B*. *longum* subspecies genomes that allowed clear distinction between *B*. *longum* subspecies (i.e. *longum* versus *infantis*) based on the presence/absence of specific genes (Table S4). Phylogenetic analysis revealed that *B*. *longum* strains within each subspecies clustered mainly according to isolation source, i.e. individual infants, rather than dietary stage (i.e. pre-weaning, weaning and post-weaning) (Figure 2B). Interestingly, strains isolated from formula-fed baby FF5 clustered into two separate clusters, irrespective of the isolation period, suggesting presence of two highly related *B*. *longum* groups within this infant. Furthermore, strains isolated from identical twins BF3 and BF4 clustered together, indicating their close relatedness.

We next sought to identify whether specific components of the *B*. *longum* subspecies pangenome were enriched in infant hosts. Each candidate gene in the accessory genome was sequentially scored according to its apparent correlation to host diet (breast vs. formula) or dietary stage. A gene annotated as α-L-arabinofuranosidase, along with four other genes coding for hypothetical proteins, were predicted to be enriched in *B*. *longum* strains isolated from breast-fed infants. Alpha-L-arabinofuranosidases are enzymes involved in hydrolysis of terminal non-reducing α-L-arabinofuranoside residues in α-L-arabinosides and act on such carbohydrates as (arabino)xylans (Ichinose et al., 2008; Ahmed et al., 2013). In addition, two genes coding for hypothetical proteins and a gene coding for mobility protein A were overrepresented in strains isolated from formula-fed infants. No associations between genes and dietary stages in *B*. *longum* nor any associations in *B*. *infantis* were observed (Table S5).

**Figure 3:**
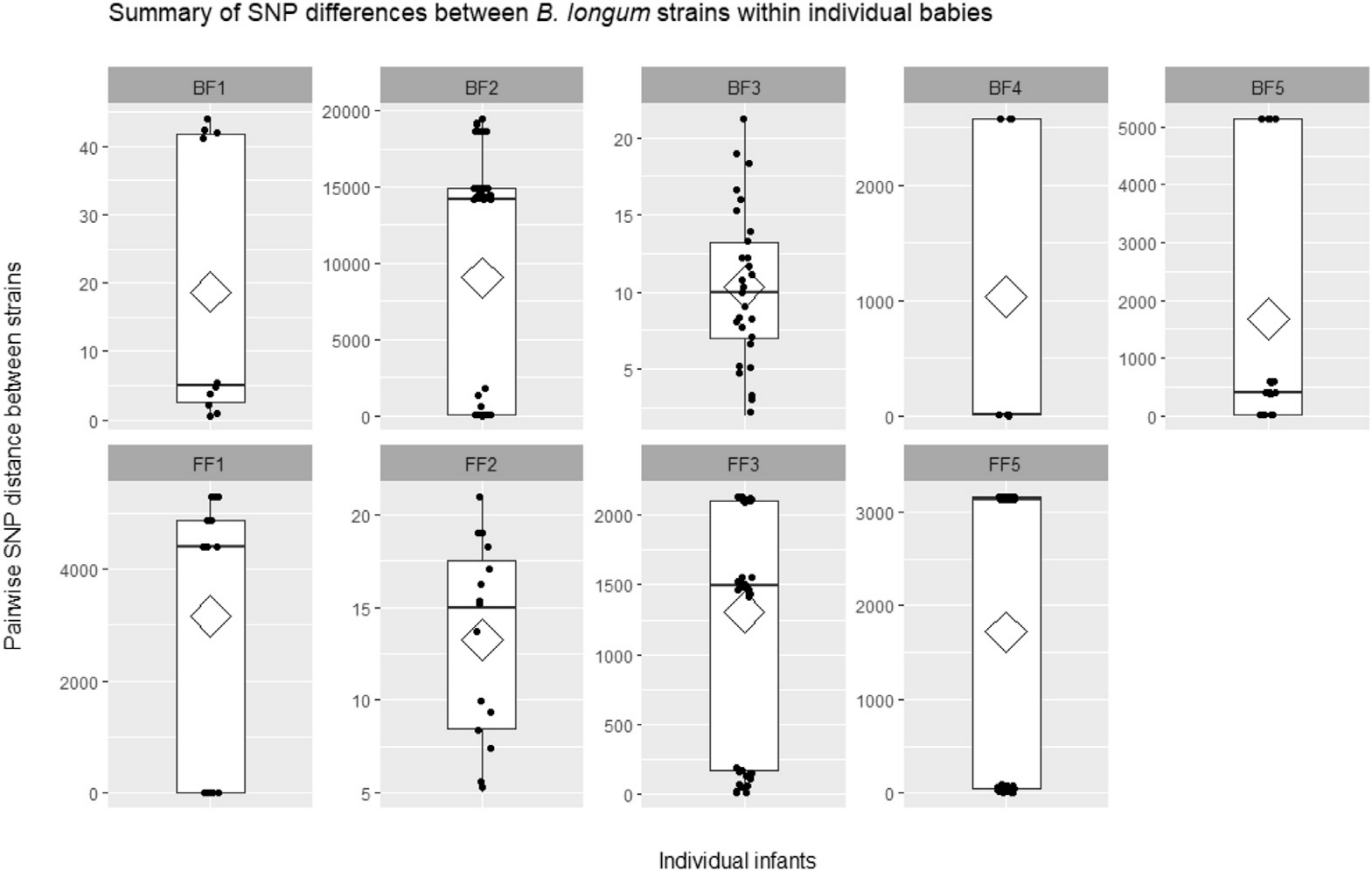
Pairwise SNP Distances between *B*. *longum* Strains of the Same Subspecies within Individual Infants Individual points show data distribution, diamonds indicate the group mean, box plots show group median and interquartile range. See also Table S6.

As our strains were isolated from individual infants at different time points, we next sought to determine their intra-strain diversity; for this we used the first *B*. *longum* isolate from each infant as the “reference” strain to which all other strains from the same infant were compared (Figure 3). Infants BF1, BF3, and FF2 had the lowest strain diversity, with respective mean pairwise SNP distances of 18.7 ± 20.3 SNPs (mean ± sd), 10.3 ± 5.0 SNPs, and 13.3 ± 5.3 SNPs. These results suggest strains isolated from these infants may be clonal, indicating long-term persistence despite dietary changes. Surprisingly, analysis of strains isolated from breast-fed identical twins BF3 and BF4 revealed higher strain diversity in baby BF4 (1034.5 ± 1327.1 SNPs), compared with the highly similar strains in infant BF3 (i.e. 10.3 ± 5.0 SNPs). Based on these results, we conducted SNP analysis on *B*. *longum* strains isolated from both babies and found that out of 13 strains analyzed (n = 8 from BF3 and n = 5 from BF4), 12 isolated during pre-weaning, weaning, and post-weaning appeared to be clonal (with mean pairwise SNP distance of 10.0 ± 5.5 SNPs) and one strain from baby BF4 isolated post-weaning was more distant, 2,595.4 ± 2.8 SNPs. The difference in strain diversity may relate to the fact that infant BF4 received a course of antibiotics during pre-weaning (Figure 1 and Tables S1 and S2) (Roger and Mccartney, 2010). Furthermore, the presence of clonal strains in both babies suggests vertical transmission of *B*. *longum* from mother to both infants, or potential horizontal transmission between babies, consistent with previous reports (Makino et al., 2011, 2013; Milani et al., 2015b; Odamaki et al., 2018). *B*. *infantis* strains isolated from infant BF2 showed the highest strain diversity of 9,030.9 ± 8,036.6 SNPs. Seven strains isolated during both pre-weaning and weaning periods appeared to be clonal, 6.3 ± 1.6 SNPs, whereas four strains isolated during weaning and post-weaning were more distant, with mean pairwise SNP distance of 14,983.5 ± 4,658.3 SNPs (Table S6).

### Functional Annotation of *B*. *longum* Subspecies Genomes—Carbohydrate Utilization

To assess genomic differences between our strains at a functional level, we next assigned functional categories to ORFs of each *B*. *longum* genome. Carbohydrate transport and metabolism was identified as the second most abundant category (after unknown function), reflecting the saccharolytic lifestyle of *Bifidobacterium* (Figure S1) (Pokusaeva et al., 2011, Milani et al., 2015a). *B*. *longum* had a slightly higher proportion of carbohydrate metabolism and transport genes (11.39 ± 0.31%) compared with *B*. *infantis* (10.20 ± 0.60%), which is consistent with previous reports (Ventura et al., 2009; Sela and Mills, 2010). *B*. *longum* strains isolated during pre-weaning had a similar proportion of carbohydrate metabolism genes in comparison with the strains isolated post-weaning: 11.28 ± 0.23% and 11.48 ± 0.38%, respectively. Furthermore, we obtained similar results for *B*. *longum* strains isolated from breast- and formula-fed infants, with respective values of 11.41 ± 0.21% and 11.38 ± 0.38%. In contrast, *B*. *infantis* strains isolated pre-weaning had a lower proportion of carbohydrate metabolism genes in their genomes compared with the ones isolated post-weaning: 9.90 ± 0.24% and 11.20 ± 0.01%, respectively (Table S7).

**Figure 4:**
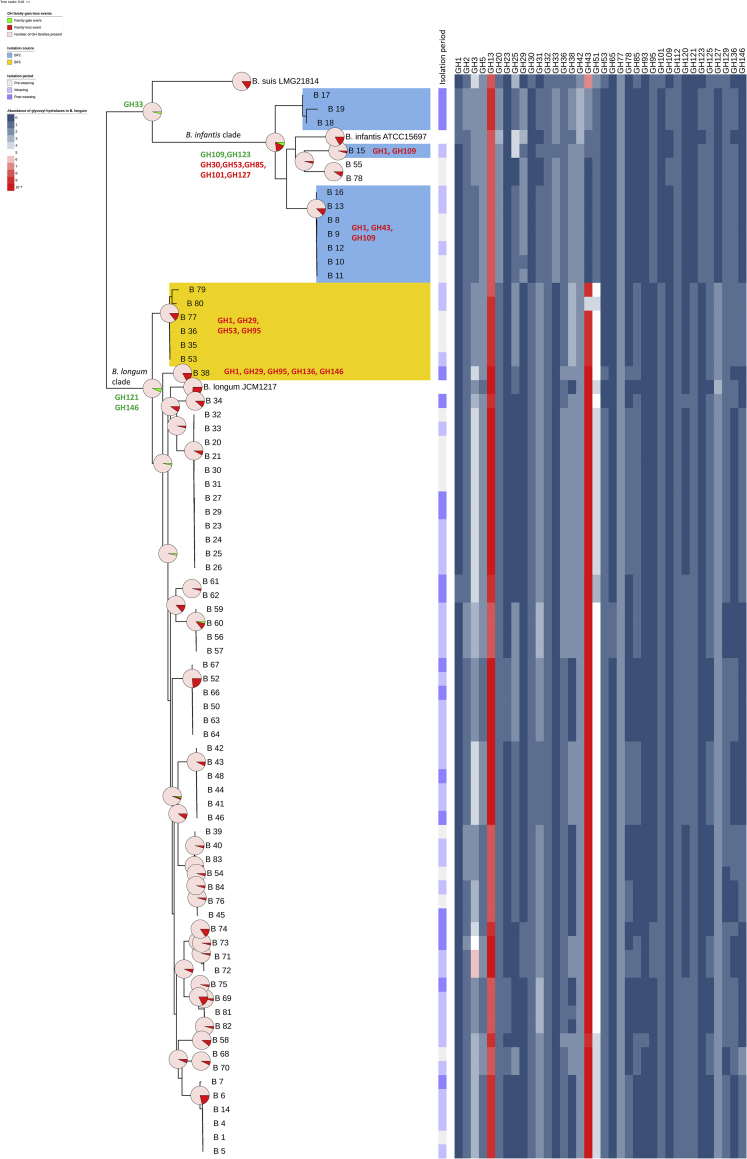
Gene-Loss Events and Abundance of GH Families within *B*. *longum* Subspecies Pie charts superimposed on the whole genome SNP tree represent predicted GH family gain-loss events within *B*. *longum* and *B*. *infantis* lineages. Due to the size of the tree, examples of detailed gain loss events have been provided for main lineages, as well as baby BF2 (strains highlighted with light blue) and BF5 (strains highlighted with yellow). Heatmap represents abundance of specific GH families predicted in analyzed *B*. *longum* strains. See also Tables S8 and S9.

One of the major classes of carbohydrate-active enzymes comprises glycosyl hydrolases (GH), which facilitate glycan metabolism in the gastrointestinal tract. We thus sought to investigate and compare the arsenal of GHs in *B*. *longum* using dbCAN2. We identified a total of 36 different GH families in all *Bifidobacterium* strains. *B*. *longum* was predicted to contain 55 GH genes per genome on average (2.72% of OFRs), whereas this number was lower for *B*. *infantis* strains, ~37 GH genes per genome (1.62% of ORFs) (Figure 4 and Table S8). The predominant GH family was GH43—enzymes involved in metabolism of complex plant carbohydrates such as (arabino)xylans (Viborg et al., 2013), followed by GH13 (starch), GH51 (hemicelluloses), and GH3 (plant glycans) (Milani et al., 2015a, 2016).

Within the *B*. *longum* group, strains isolated during pre-weaning had a slightly lower mean number of GH genes compared with strains isolated post-weaning (54.46 ± 2.81 vs. 56.85 ± 2.77). Moreover, strains isolated from breast-fed babies contained an average of 53.96 ± 3.82 GH genes per genome, whereas this number was slightly higher for strains isolated from formula-fed infants—56.47 ± 2.96. Further analysis revealed that these differences appeared to be intra-host-specific and diet-related. For example, strains isolated from breast-fed twins BF3 and BF4 pre-weaning had 11 GH43 genes per genome, whereas the pre-weaning strain from formula-fed baby FF3 had 13 GH genes per genome predicted to belong to this GH family. Similarly, strains isolated from babies BF3 and BF4 post-weaning had 11 predicted GH genes, whereas the three strains isolated from infant FF3 were predicted to contain 16, 16, and 18 GH genes per genome, respectively (Table S8).

We next determined if these GH genes differences statistically correlated with breast- and formula-fed groups (Table S8). Significant differences (p < 0.05) were observed between mean numbers of GH genes belonging to the predominant GH families (GH43—higher abundance in FF babies, GH13—higher abundance in BF babies, and GH51—higher abundance in FF babies), and several other GH families, including GH5 (β-glucosidases and β-mannosidases), GH38 (mannosylglycerate hydrolases), and GH36 (α-galactosidases), all more abundant in BF babies. Further analysis of dietary phases suggested significant differences in GH genes between breast- and formula-fed groups during pre-weaning (e.g. families GH43, GH13, GH5, GH38) but not in the post-weaning phase (Table S8).

Because glycosyl hydrolases belonging to distinct GH families may have similar catalytic properties, we next grouped the GH genes for which the predicted enzyme class annotation was available and investigated their abundance (Table S9). The predominant enzyme classes in *B*. *longum* strains were non-reducing end α-L-arabinofuranosidases belonging to GH43 and GH51, followed by β-galactosidases (GH2 and GH42), oligo-1,6-glucosidases (GH13), and β-*N-*acetylhexosaminidases (GH3 and GH20).

The mean numbers of enzyme classes between breast- and formula-fed babies significantly differed (p < 0.05) in the top three above-mentioned predominant enzyme classes as well as several other less abundant ones, including non-reducing end β-L-arabinofuranosidases (GH127 and GH146—higher abundance in BF babies), α-galactosidases (GH36—higher abundance in BF babies), and endo-1,5-α-L-arabinases (GH43—higher abundance in FF babies). Additional analysis of dietary phases indicated significant differences between breast- and formula-fed groups during pre-weaning (e.g. non-reducing end α-L-arabinofuranosidases, β-galactosidases, oligo-1,6-glucosidases as well as α-galactosidases) but not during post-weaning (Table S9).

We next examined the predicted glycosyl hydrolase repertoire of *B*. *infantis* strains, with the caveat that the majority of the strains belonging to this subspecies were isolated from a single infant. In contrast to the *B*. *longum* group, the most abundant GH family was GH13 (starch), followed by GH42, GH20, and GH38 (Table S8). *B*. *infantis* strains also harbored genes predicted to encode members of the GH33 family, which contains exo-sialidases (Milani et al., 2015a). Strains isolated pre-weaning were predicted to contain an average of 34.83 ± 0.4 GH genes per genome, whereas this number was higher for the strains isolated post-weaning (i.e. 43.00 ± 0.00 GH genes). *B*. *infantis* strains isolated post-weaning contained families GH1 and GH43 that were absent in the strains isolated pre-weaning. The GH1 family contains enzymes such as β-glucosidases, β-galactosidases, and β-D-fucosidases active on a wide variety of (phosphorylated) disaccharides, oligosaccharides, and sugar–aromatic conjugates (Suzuki et al., 2013). The analysis of enzyme classes in the *B*. *infantis* strains suggested that β-galactosidases (GH2 and GH42) were predominant in this group, followed by β-*N*-acetylhexoaminidases (GH3 and GH20), 4-α-glucanotransferases (GH77), and oligo-1,6-glucosidases (GH13) (Table S9).

Members of the genus *Bifidobacterium* have previously been shown to contain GH genes involved in metabolism of various HMOs present in breast milk (Garrido et al., 2015, 2016). We identified genes belonging to GH29 and GH95 (α-L-fucosidases found active on fucosylated HMOs (Sela et al., 2012; Garrido et al., 2016)) in all our *B*. *infantis* strains, as well as four *B*. *longum* strains isolated from formula-fed baby FF3. Furthermore, we found GH20 and GH112 genes (lacto-*N*-biosidases and galacto-*N*-biose/lacto-*N*-biose phosphorylases shown to be involved in degradation of isomeric lacto-*N*-tetraose (LNT) (Kitaoka, 2012)) in all our *B*. *infantis* and *B*. *longum* strains (Table S8).

Overall, these findings suggest differences in general carbohydrate utilization at different stages suggesting adaptation of *Bifidobacterium* to a changing early life nutritional diet, which may be a factor facilitating establishment of these bacteria within individuals during infancy.

### Prediction of Gain and Loss of GH Families in *B*. *longum*

Given the differences in the carbohydrate utilization profiles between *B*. *longum* and *B*. *infantis*, we next investigated the acquisition and loss of GH families. For this purpose, we additionally predicted the presence of GH families in type strains *B*. *longum* subsp. *longum* JCM 1217^T^, *B*. *longum* subsp. *infantis* ATCC 15697^T^, and *B*. *longum* subsp. *suis* LMG 21814^T^ with dbCAN2 and generated a whole genome SNP tree to reflect gene loss/gain events more accurately (Figure 4 and Table S10). Both *B*. *longum* and *B*. *infantis* lineages appear to have acquired GH families (when compared to the common ancestor of the phylogenetic group), with the *B*. *longum* lineage gaining two GH families (GH121 and GH146) and the *B*. *infantis* lineage one GH family (GH33). Within the *B*. *infantis* lineage, which also contains the *B*. *suis* type strain, the *B*. *infantis* taxon has further acquired two and lost five GH families. These findings suggest that the two human-related subspecies have followed different evolutionary paths, which is consistent with our observation of differences between *B*. *longum* and *B*. *infantis* resulting from phylogenomic analyses. Intriguingly, strain adaptation to the changing host environment (i.e. individual infant gut) seems to be driven by loss of specific GH families (Figure 4). For example, *B*. *infantis* strains isolated during pre-weaning and weaning from baby BF2 appear to be missing up to three GH families (GH1, GH43, and GH109) present in strains isolated post-weaning. Lack of family GH43 (containing enzymes involved in metabolism of a variety of complex carbohydrates, including plant-derived polysaccharides) in early life *B*. *infantis* strains may explain nutritional preference of this subspecies for an HMO-rich diet. Similarly, we observed differential gene loss events in *B*. *longum* strains from individual hosts. For example, all strains isolated from baby BF5 appear to lack GH families GH1, GH29, and GH95. However, strains isolated pre-weaning additionally lacked GH53 family, which includes endogalactanases shown to be involved in liberating galactotriose from type I arabinogalactans in *B*. *longum* (Hinz et al., 2005). In contrast, strain B_38 isolated from this infant (BF5) post-weaning appears to have lost families GH136 and GH146. Interestingly, members of family GH136 are lacto-*N*-biosidases responsible for liberating lacto-*N*-biose I from LNT, an abundant HMO unique to human milk (Yamada et al., 2017), whereas family GH146 contains β-L-arabinofuranosidases displaying *exo*-activity on β-linked arabinofuranosyl groups. These events may be linked to dietary changes (withdrawal of breast milk) and/or a shift in the composition of the microbiota post-weaning. Only one *B*. *longum* strain was isolated post-weaning from this baby; however, FISH analysis (Figure 1 and Table S1) revealed an increase in the bacteroides group, which might explain the loss of family GH146 by strain B_38 as the founding member of GH146 family, β-L-arabinofuranosidase, was first characterized in *Bacteroides thetaiotaomicron* (Luis et al., 2018). Overall, the presence of intra-individual and strain-specific GH family repertoires in *B*. *longum* suggests their adaptation to host-specific diet. The presence of strains with different GH content at different dietary stages further indicates potential acquisition of new *Bifidobacterium* strains with nutrient-specific adaptations in response to the changing infant diet.

### Prediction of Single Nucleotide Polymorphisms in Glycosyl Hydrolases

Given the intra-strain diversity in the nine babies and the differences in GH repertoires between *B*. *longum* and *B*. *infantis*, we next sought to examine nucleotide-level differences in glycosyl hydrolase genes between strains in individual infants (Table S11). Unsurprisingly, we did not identify any significant SNPs that may lead to functional changes in GH genes in infants that had the lowest strain diversity (infants BF1, BF3 and FF2) (Table S6). The highest number of GH genes with predicted variants was recorded for *B*. *infantis* strains from baby BF2. In total, 52 synonymous variants and 29 missense variants were predicted at 81 different positions in 12 GH genes across strains that showed the highest diversity from the first “reference” isolate, namely one strain isolated during weaning and the three strains isolated post-weaning. Several missense variants, both complex and single, were recorded at several positions in the predominant enzyme classes, i.e. β-galactosidases (EC 3.2.1.23) and β-*N*-hexosaminidases (EC 3.2.1.52).

Similarly, both synonymous and missense variants were predicted in *B*. *longum* strains less closely related to “reference” strains from breast-fed (BF4 and BF5) and formula-fed (FF1, FF3 and FF5) babies. We did not observe any trend in the distribution of SNPs across GH genes in *B*. *longum* strains. The number of predicted variants, the number of GH genes with identified mutations, and their enzyme classification differed between individual infants. For example, in baby BF4 9 out of 10 predicted variants (4 synonymous and 5 missense) were identified in an α-xylosidase in a strain isolated post-weaning, whereas in baby FF5 14 synonymous and 10 missense variants were predicted at 24 positions in 7 different GH genes across strains isolated during weaning and post-weaning. Some missense changes do not compromise normal protein function, whereas others can change essential aspects of protein maturation, activity, or stability (Miosge et al., 2015). The presence of missense variants in GH genes of *B*. *longum* strains may indicate potential functional differences and provide additional explanation to intra-strain and intra-individual carbohydrate metabolism profiles of these bacteria; however, experimental evidence would be essential to confirm the importance of these predictions.

### Phenotypic Characterization of Carbohydrate Utilization

**Figure 5:**
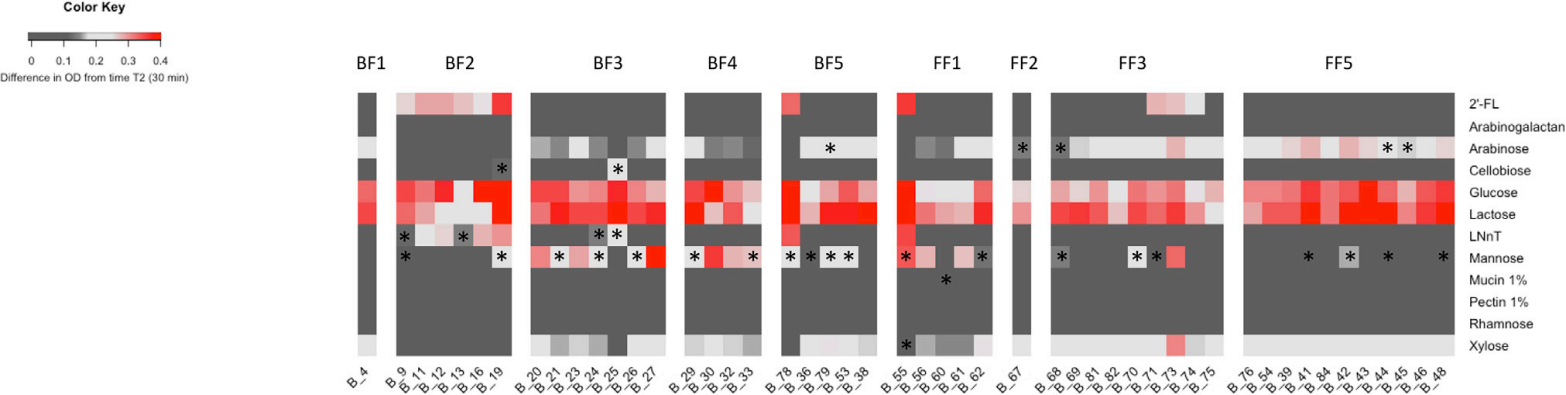
Growth Performance of *B*. *longum* Strains on Different Carbon Sources Heatmap displays the difference in average growth of triplicates between T_2_ (30 min) and T_end_ (48 hr). Moderate growth is considered above 0.15 difference in OD from time T_2_, high growth above 0.25 difference in OD from time T_2_. Asterisks represent strains for which inconsistent growth was recorded (difference in OD of at least 0.15 between any of the duplicates in the triplicate experiment). See also Table S12.

*Bifidobacterium longum* has previously been shown to metabolize a range of carbohydrates, including dietary and host-derived glycans (Watson et al., 2013; Arboleya et al., 2018). Given the predicted differences in carbohydrate metabolism profiles and to understand strain-specific nutrient preferences, we next determined their glycan fermentation capabilities. We performed growth assays on 49 representative strains from all nine infants, cultured in modified MRS supplemented with selected carbohydrates as the sole carbon source. For these experiments, we chose both plant- and host-derived glycans that we would expect to constitute components of the early life infant diet (Mills et al., 2019). Although all *B*. *longum* strains were able to grow on simple carbohydrates (i.e. glucose and lactose), we also observed subspecies-specific complex carbohydrate preferences, consistent with bioinformatic predictions (Figure 5). To represent host-derived carbohydrates, we selected 2′-fucosyllactose (2′-FL) and lacto-*N*-neotetraose (LNnT) as examples of HMOs found in breast milk. Out of the tested isolates, all *B*. *infantis* strains were able to metabolize 2′-FL, as were three *B*. *longum* strains isolated from a formula-fed baby FF3 during weaning and post-weaning (Figure 5). These results supported the computational analysis and the identification of genes potentially involved in degradation of fucosylated carbohydrates in the genomes of these isolates (GH29 and GH95). Although bioinformatics identified the presence of genes involved in metabolism of isomeric LNT in all our strains (GH20 and GH112), LNnT metabolism in *B*. *infantis* was strain specific, with most strains showing what we considered moderate (above 0.15 difference in OD from time T_2_) to high growth rates (above 0.25 difference in OD from time T_2_), with two strains displaying inconsistent growth (Table S12). Out of *B*. *longum* strains, B_24 and B_25 (isolated during weaning from breast-fed baby BF3) also showed growth on LNnT, albeit this was inconsistent. In contrast to all other *B*. *longum* strains, strain B_25 was not able to metabolize plant-derived arabinose and xylose despite the predicted presence of genes involved in metabolism of monosaccharides (GH43, GH31, GH2). However, it was one of the two strains (out of 49 tested) that showed growth on cellobiose in 2/3 experiments, the other one being the post-weaning *B*. *infantis* strain B_19 isolated from baby BF2. Given these interesting results, we performed additional assays using cellobiose as the sole carbon source over 72 h, in which the *B*. *longum* strain B_25 showed high growth rate (above 0.25 difference in OD from time T_2_), whereas the *B*. *infantis* B_19 strain did not grow at all (Table S12). In addition, both *B*. *longum* and *B*. *infantis* strains showed varying degrees of growth performance on mannose, even when analyzing the same strain, whereas none of the tested strains were able to grow on arabinogalactan, pectin, or rhamnose (Figure 5).

**Figure 6:**
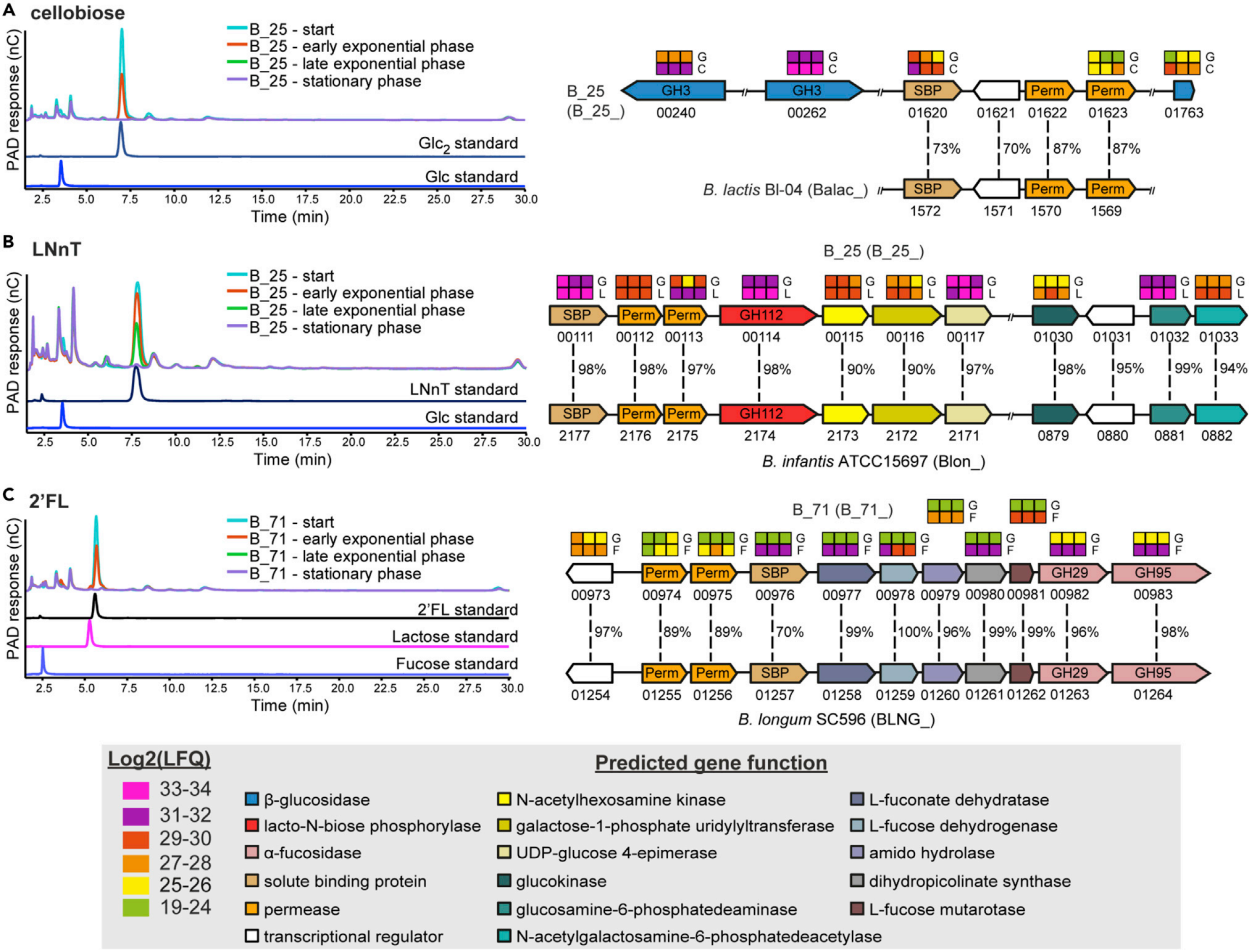
Carbohydrate Uptake Analysis and Proteomics of *B. longum* Strains B_25 and B_71 HPAEC-PAD traces showing mono-, di-, and oligo-saccharides detected in the supernatant of either B_25 or B_71 single cultures during growth in mMRS supplemented with (A) cellobiose; (B) LNnT; (C) 2′-FL. The data are representative of biological triplicates. Abbreviations: LNnT, Lacto-*N*-neotetraose; Glc, glucose; Glc2, cellobiose; 2′-FL, 2′-fucosyllactose. Panel on the right shows (A) cellobiose; (B) LNnT; (C) 2′-FL utilization clusters in B_25 and B_71 and proteomic detection of the corresponding proteins during growth on HMOs. Heatmaps above genes show the LFQ detection levels for the corresponding proteins in triplicates grown on glucose (G); cellobiose (C); LNnT (L); and 2′-FL (F). Numbers between genes indicate percent identity between corresponding genes in homologous gene clusters relative to strains B_25 and B_71. Numbers below each gene show the locus tag in the corresponding genome. Locus tag numbers are abbreviated with the last numbers after the second hyphen (for example B_25_XXXXX). The locus tag prefix for each strain is indicated in parenthesis beside the organism name. See also Tables S13 and S14.

To further characterize strains identified above for putative carbohydrate degradation genes, we performed carbohydrate uptake analysis and proteomics. *B*. *longum* strain B_25, from one of the breast-fed identical twins that showed growth on LNnT and cellobiose, and formula-fed strain B_71, which was able to grow on 2′-FL, were chosen. Supernatant from these cultures was initially subjected to high-performance anion-exchange chromatography (HPAEC) to evaluate the carbohydrate-depletion profiles (Figure 6). In all three cases, the chromatograms showed complete utilization of the tested carbohydrates and absence of any respective degradation products in the stationary phase culture. The depletion of cellobiose by B_25 and 2′-FL by B_71 occurred in the early exponential phase, whereas LNnT was still detected in the culture supernatant until the late exponential phase of growth, suggesting that cellobiose and 2′-FL were internalized more efficiently than LNnT. We next determined the proteome of B_25 and B_71 when growing on cellobiose, LNnT and 2′-FL compared with glucose (Figures 6A–6C and Table S13). The top 10 most abundant proteins in the cellobiose proteome of B_25 included three β-glucosidases belonging to GH3 family, as well as a homologue of transport gene cluster previously shown to be upregulated in *B*. *animalis* subsp. *lactis* Bl-04 during growth on cellobiose (Figure 6A and Table S14) (Andersen et al., 2013). Among the three β-glucosidases, B_25_00240 showed 98% sequence identity to the structurally characterized BlBG3 from *B*. *longum*, which has been shown to be involved in metabolism of the natural glycosides saponins (Yan et al., 2018). B_25_01763 and B_25_00262 showed 46% identity to the β-glucosidase Bgl3B from *Thermotoga neapolitana* (Pozzo et al., 2010) and 83% identity to BaBgl3 from *B*. *adolescentis* ATCC 15703 (Florindo et al., 2018), respectively, two enzymes previously shown to hydrolyze cello-oligosaccharides. With respect to LNnT metabolism by the same strain, the most abundant proteins were encoded by genes located in two gene clusters (B_25_00111–00117 and B_25_00130-00133) with functions compatible with LNnT import, degradation to monosaccharides, and further metabolism. The gene clusters contain the components of an ABC-transporter (B_25_00111–00113), a predicted intracellular GH112 lacto-*N*-biose phosphorylase (B_25_00114), an *N*-acetylhexosamine 1-kinase (B_25_00115) and enzymes involved in the Leloir pathway. All these proteins were close homologues to proteins previously implicated in the degradation of LNT/LNnT by type strain *B*. *infantis* ATCC 15697^T^ (Ozcan and Sela, 2018) (Figure 6B and Table S14). Interestingly, all clonal strains isolated from twin babies BF3 and BF4 also contained close homologues of all the above-mentioned genes in their genomes, in some cases identical to those determined in B_25; however, only strain B_25 was able to grow on cellobiose and LNnT. Growth of B_71 on 2′-FL corresponded to increased abundance of proteins encoded by the gene cluster B_71_00973-00983. These proteins showed close homology to proteins described for *B*. *longum* SC596 and included genes for import of fucosylated oligosaccharides, fucose metabolism, and two α-fucosidases belonging to the families GH29 and GH95 (Figure 6C and Table S14) (Garrido et al., 2016).

## Discussion

High abundance of *Bifidobacterium*, and *B*. *longum* in particular, in early infancy is strongly linked to availability of nutrients (Koenig et al., 2011; Backhed et al., 2015; Yamada et al., 2017). In this study, we aimed to investigate the adaptations of *B*. *longum* to the changing infant diet during the early life developmental window. Profiling microbiota composition data (Roger and Mccartney, 2010), genomic diversity of 75 *B*. *longum* strains (isolated from infants at different dietary stages), and growth performance on different carbohydrates revealed intra-individual and diet-related differences, which links to strain-level metabolism properties for specific dietary components.

The FISH results corroborate findings of previous studies investigating the infant gut microbiota—inter-individual variability during pre-weaning and weaning, with a shift toward a more adult-like fecal microbiota associated with more complex diet at post-weaning across all samples (Koenig et al., 2011, Mckeen et al., 2019). *Bifidobacterium* constituted the predominant group in breast-fed infants during pre-weaning and weaning, whereas the composition of microbiota of the formula-fed infants during these stages was more complex.

Our comparative genomic analysis indicates that clonal strains of *B*. *longum* can persist in individuals through infancy, for at least 18 months, despite significant changes in diet during weaning, which is consistent with previous reports (Maldonado-Gomez et al., 2016; Odamaki et al., 2018). Concurrently, new strains (that display different genomic content and potential carbohydrate metabolism capabilities) can be acquired, possibly in response to the changing diet. Previously, strain shift in relation to withdrawal of breast milk has been suggested for *B*. *infantis* by Vatanen et al. (2019) based on strain-level metagenomic approach. Similarly, Asnicar et al. (2017) suggested that originally acquired maternal strains of *B*. *longum* can be replaced at later life stages. Initial vertical acquisition of *Bifidobacterium* from mother to newborn babies has been well documented (Mikami et al., 2012; Makino et al., 2013; Milani et al., 2015b; Asnicar et al., 2017); however, details of strain transmission events in later life are currently unclear. Work of Odamaki et al. (2018) suggested person-to-person horizontal transmission of a particular *B*. *longum* strain between members of the same family, with direct transfer, common dietary sources, or environmental reservoirs, such as family homes (Lax et al., 2014) acting as potential vehicles and routes for strain transmission. Our results showed the presence of clonal strains in identical twins BF3 and BF4, which may have resulted from maternal transfer. However, potential strain transmission between these infants living in the same environment may also occur. Wider studies involving both mothers and twin babies (and other siblings) could provide details on the extent, timing, and location of transmission events between members of the same household.

Another aspect of comparative genomic analysis involved *in silico* prediction of genes belonging to GH families. This analysis revealed genome flexibility within *B*. *longum*, with differences in GH family content between strains belonging to the same subspecies as described previously; *B*. *infantis* predominantly enriched in GH families implicated in the degradation of host-derived breast milk-associated dietary components such as HMOs and *B*. *longum* containing GH families involved in the metabolism of plant-derived substrates (Milani et al., 2015a; Milani et al., 2016). Previously, Vatanen et al. (2019) suggested that the presence of the HMO gene cluster allowing for intracellular HMO utilization in *B*. *infantis* strains, in particular, confers a competitive advantage leading to higher relative abundance of this subspecies in the early life microbiota. Our analysis of *B*. *infantis* group identified the presence of glycosyl hydrolases associated with HMO degradation in all isolates and revealed subspecies-specific differences in GH content between pre- and post-weaning strains. Moreover, we observed differences in the number of genes belonging to the most abundant GH families (e.g. GH43) between breast-fed and formula-fed strains at different dietary stages, which can be linked to nutrient availability. Surprisingly, we computationally and phenotypically identified closely related weaning and post-weaning *B*. *longum* strains capable of metabolizing HMOs (i.e. 2′-FL) in a formula-fed baby that only received standard non-supplemented (i.e. no prebiotics or synthetic HMOs) formula. The analysis of SNP variants in genes identified as glycosyl hydrolases predicted the presence of missense mutations in both *B*. *longum* and *B*. *infantis* strains. Given that some missense variants can compromise protein function (Miosge et al., 2015), our results suggest potential functional differences that could further explain intra-strain and intra-individual carbohydrate metabolism profiles of *B*. *longum*. However, experimental validation would be essential to confirm the importance of variant predictions.

Recorded phenotypic data support the results of genomic analyses and further highlight differences in carbohydrate utilization profiles between and within *B*. *longum* and *B*. *infantis*. As highlighted above, the ability of *B*. *infantis* to grow on different HMOs may facilitate their early life establishment. Similarly, *B*. *longum* preference for plant-based nutrients may influence their ability to persist within individual hosts through significant dietary changes. Differential growth of strains that are genotypically similar on various carbohydrate substrates and the ability of formula-fed strains to metabolize selected HMOs suggest that *Bifidobacterium* possess an overall very broad repertoire of genes for carbohydrate acquisition and metabolism that may be differentially switched on and off in response to the presence of specific dietary components (Dworkin and Losick, 2001; Slager and Veening, 2016). Another explanation for these results may be a potential influence of the intra-individual environment on epigenetic mechanisms in these bacteria. One potential factor involved in this process may be a cooperative effort supported by cross-feeding activities among *Bifidobacterium* or between *Bifidobacterium* and other members of the early life microbiota, e.g. *Bacteroides* and *Eubacterium* species (Rios-Covian et al., 2013; Milani et al., 2015a; Schwab et al., 2017; Lawson et al., 2020). Indeed, the FISH analysis revealed the presence of bacteria detected by probes Bac303 (bacteroides) and ER482 (eubacterium) in fecal samples of both breast- and formula-fed infants, with intra-individual variation at different dietary stages. Although *B*. *infantis* is principally known as a specialist HMO degrader, we did note growth of one of the *B*. *infantis* strains from formula-fed baby FF1 on xylose. However, this growth profile was not consistent between experiments and therefore we did not pursue a fuller characterization. However, future examination of the ability of *B*. *longum* subsp. *infantis* to degrade a wider range of non-HMO carbohydrate sources in early life could provide additional insight into carbohydrate metabolism properties of this subspecies and its role in ecosystem structuring during transition to a more complex diet.

Glycan uptake analysis and proteomic investigation allowed us to determine mechanisms that selected *B*. *longum* strains to metabolize different carbohydrates. A common feature, based on the predicted activity of the most abundant proteins detected during grown on the three substrates (cellobiose, LNnT and 2′-FL), was that they were all imported and “selfishly” degraded intracellularly, therefore, limiting release of degradation products that could allow cross-feeding by other gut bacteria. This is in line with the carbohydrate uptake analysis, where no peak for cellobiose, LNnT, and 2′-FL degradation products could be detected. Cellobiose uptake in B_25 occurs via a mechanism similar to *B*. *animalis* subsp. *lactis* Bl-04 (*B*. *lactis*) (Andersen et al., 2013); cellobiose hydrolysis appears to be mediated by the activity of three intracellular β-glucosidases, although further confirmatory biochemical characterization of these enzyme is still required. B_25 was observed to utilize LNnT using a pathway similar to that described in *B*. *longum* subsp. i*nfantis* whereby LNnT is internalized via an ABC-transporter (B_25_00111-00113) followed by intracellular degradation into constituent monosaccharides by a GH112 (B_25_00114) and an *N*-acetylhexosamine 1-kinase (B_25_00115). LNnT degradation products are further metabolized to fructose-6-phosphate by activities that include B_25_00116-00117 (galactose-1-phosphate uridyltransferase, UDP-glucose 4-epimerase, involved in the Leloir pathway) and B_25_01030-01033 (for metabolism of *N*-acetylgalactosamine) prior to entering the *Bifidobacterium* genus-specific fructose-6-phosphate phosphoketolase (F6PPK) pathway (Ozcan and Sela, 2018). B_71 is predicted to deploy an ABC-transporter (B_71_00974-00976) that allows uptake of intact 2′-FL that is subsequently hydrolyzed to L-fucose and lactose by the two predicted intracellular α-fucosidases GH29 (B_71_00982) and GH95 (B_71_00983). L-fucose is further metabolized to L-lactate and pyruvate, via a pathway of non-phosphorylated intermediates that include activities of L-fucose mutarotase (B_71_00981), L-fucose dehydrogenase (B_71_00978), and L-fuconate hydrolase (B_71_00977) as previously described for *B*. *longum* subsp. *longum* SC596 (Garrido et al., 2016). Considering that the proteins encoded by the aforementioned genes are located in the cellobiose, LNnT and 2′-FL gene clusters that share high similarity and similar organization with those found in equivalent systems in other *B*. *longum* and *B*. *lactis*, it is reasonable to suggest that the gene clusters are related and may be the results of horizontal gene transfer events between *B*. *longum*/*B*. *lactis* members residing in the infant gut microbiota. Collectively, these data reflect inter- and intra-host phenotypic diversity of *B*. *longum* strains in terms of their carbohydrate degradation capabilities and suggest that intra-individual environment may influence epigenetic mechanisms in *Bifidobacterium*, resulting in differential growth on carbohydrate substrates.

In conclusion, this research provides new insight into distinct genomic and phenotypic abilities of *B*. *longum* species and strains isolated from the same individuals during the early life developmental window by demonstrating that subspecies- and strain-specific differences between members of *B*. *longum* sp. in infant hosts can be correlated to their adaptation at specific age and diet stages.

### Limitations of the Study

Here, we used a combination of bioinformatic approaches and experimental techniques to assess genomic and phenotypic abilities of *B*. *longum* species and strains isolated during the early life developmental window. This study, however, is not without its limitations. One important caveat is the small number of *B*. *infantis* strains (n = 13) available for analysis and the fact that most of these strains (n = 11) were isolated from a single breast-fed baby (BF2). The examination of these strains provides important insight into the properties of *B*. *infantis* during the transition from breastfeeding to more diversified diet; however, it is difficult to assess how representative these results are of wider population. In addition, only one strain isolated from a formula-fed baby was identified as *B*. *infantis*, making it impossible to examine properties of members of this subspecies within this dietary group and make comparisons with breast-fed strains. Another important limitation is the fact that our strain collection only contains one bacterial strain per time point. Inclusion of additional strains could contribute further observations on inter-individual diversity of *Bifidobacterium* in infant hosts and their functional properties. To examine bacterial communities in fecal samples, we revisited and reanalyzed the data generated using FISH, but this technique has a detection limit (~10^6^ bacterial cells (wet weight feces)^−1^) (Roger and Mccartney, 2010). Thus, FISH allows investigation of important bacterial groups, but fecal samples may contain several organisms at levels below the methodological detection threshold. In addition, this technique does not allow for tracking species-level changes. This limitation could be addressed by the use of comprehensive sequencing methods, such as shotgun metagenomics, combined with advanced computational methods to achieve strain-level resolution. Furthermore, phenotypic investigation of carbohydrate metabolism properties of *B*. *longum* revealed inconsistencies in growth of individual strains on certain carbohydrates, including LNnT, cellobiose, and mannose, and we therefore only explored reproducible findings further with proteomics. Previously, variability in growth of *B*. *longum* on mannose, even when analyzing the same strain (*Bifidobacterium longum* NCC2705) has been reported (Parche et al., 2007; Liu et al., 2011). Finally, no metadata on complementary foods during weaning and infant diet post-weaning were available. This information could allow bioinformatic predictions of carbohydrate degradation properties of *B*. *longum* to be related to the specific dietary components present in weaning infant foods. Future longitudinal studies could be designed to include these data.

### Resource Availability

#### Lead Contact

Further information and requests for resources and reagents should be directed to and will be fulfilled by the Lead Contact, Lindsay J. Hall (lindsay.hall@quadram.ac.uk).

#### Materials Availability

This study did not generate new unique reagents.

#### Data and Code Availability

The draft genomes of 75 *B*. *longum* isolates have been deposited to GOLD database at https://img.jgi.doe.gov. The accession number for the draft genomes reported in this paper is GOLD: Gs0145337.

The proteomics data have been deposited to the ProteomeXchange Consortium (http://proteomecentral.proteomexchange.org) via the partner repository with dataset identifier (PRIDE). The accession number for the proteomics data reported in this paper is PRIDE: PXD017277.

## Methods

All methods can be found in the accompanying Transparent Methods supplemental file.

## Supplemental Information

Document S1. Transparent Methods and Figure S1

Table S1. FISH Probes Used to Enumerate Bacterial Groups in Infants (Adapted from Roger & McCartney (2010)), Proportional Representation of FISH Data for Each Baby, Summative Analysis of Bacterial Populations in Relation to Dietary Phases (Separate Tabs), Related to Figure 1

Table S2. Demographic Profiles of Infants in This Study, Genomic Data on the Isolates In This Study, List of Publicly Available Genomes Used in This Study (Separate Tabs), Related to Figure 2

Table S3. Average Nucleotide Identity Matrix for the Isolates Used in This Study, Related to Figure 2

Table S4. Gene Presence-Absence Matrix for the *B. longum* Isolates Used in This Study, Related to Figure 2

Table S5. List of Overrepresented Genes Associated with Breast- and Formula-Feeding Detected Using Scoary (Separate Tabs), Related to Figure 2

Table S6. Pairwise SNP Distance Matrix between Isolates within Individual Infants, Related to Figure 3

Table S7. Summary of COG Categories Identified in *B. longum Isolates,* Related to Figure 4 COG categories legend, COG categories identified in all *B. longum* samples, as well as in specific groups: *B. infantis*, *B. longum*, isolates from breast-fed infants, isolates from formula-fed infants (separate tabs)

Table S8. Summary of GH Families Identified in all *B. longum* Isolates, as well as in Specific Groups: *B. longum* across all Dietary Phases, *B. longum* Pre-weaning, *B. longum* Post-weaning, *B. longum* Isolates from Breast-Fed Infants, *B. longum* Isolates from Formula-Fed Infants, Comparison between *B. longum* Breast- and Formula-Fed Groups, *B. infantis, B. infantis* Pre-weaning, *B. infantis* Post-weaning (Separate Tabs), Related to Figure 4

Table S9. Summary of Enzyme Classes Identified in all *B. longum* Isolates, as well as in Specific Groups: *B. longum* across All Dietary Phases, *B. longum* Pre-weaning, *B. longum* Post-weaning and *B. infantis* (Separate Tabs), Related to Figure 4

Table S10. Prediction of Glycosyl Hydrolase Family Gain-Loss Events in *B. longum* Isolates, Related to Figure 4

Table S11. Summary of SNPs in Predicted Glycosyl Hydrolases in Breast-Fed and Formula-Fed Babies, Related to Figures 3 and 4

Table S12. Results of Growth Experiments Performed for selected *B. longum* and *B. infantis* Strains from Nine Babies, Related to Figure 5 Strains (1%v/v) were grown in modified (m)MRS (pH 6.8) supplemented with cysteine HCl at 0.05% and 2% (w/v) of selected carbohydrates, except for pectin and mucin (1%). Experiments were performed in triplicates; the data are presented as difference in OD between T_2_ (30min) and T_end_ (48h) for growth on 12 selected carbohydrate sources and T_2_ (30min) and T_end_ (72h) for an additional experiment on cellobiose (separate tabs)

Table S13. Proteomics Data from Growth of Strain B_25 on LNnT and Cellobiose and Strain B_71 on 2′-FL (Separate Tabs), Related to Figure 6

Table S14. Homology of Proteins Identified by Proteomics in Strain B_25 Grown on Cellobiose and LNnT and Strain B_71 Grown on 2′-FL to Known *Bifidobacterium* Gene Clusters (BLAST+, e-value 1e-50) (Separate Tabs), Related to Figure 6
