# Supplementary material for: Succession of Bifidobacterium longum Strains in Response to a Changing Early Life Nutritional Environment Reveals Dietary Substrate Adaptations
Source: iScience. 2020 Jul 15;23(8):101368. doi: 10.1016/j.isci.2020.101368 (PMC7390879; doi:10.1016/j.isci.2020.101368)
Supplement: Document S1. Transparent Methods and Figure S1 [file mmc1.pdf]

iScience, Volume *23*

Supplemental Information

Succession of *Bifidobacterium longum* Strains in Response to a Changing Early Life Nutritional Environment Reveals Dietary Substrate Adaptations

Magdalena Kujawska, Sabina Leanti La Rosa, Laure C. Roger, Phillip B. Pope, Lesley Hoyles, Anne L. McCartney, and Lindsay J. Hall

Supplemental Figures

Figure S1. COG classification of the proteins encoded by *B. longum* strains. Related to Figure 4.

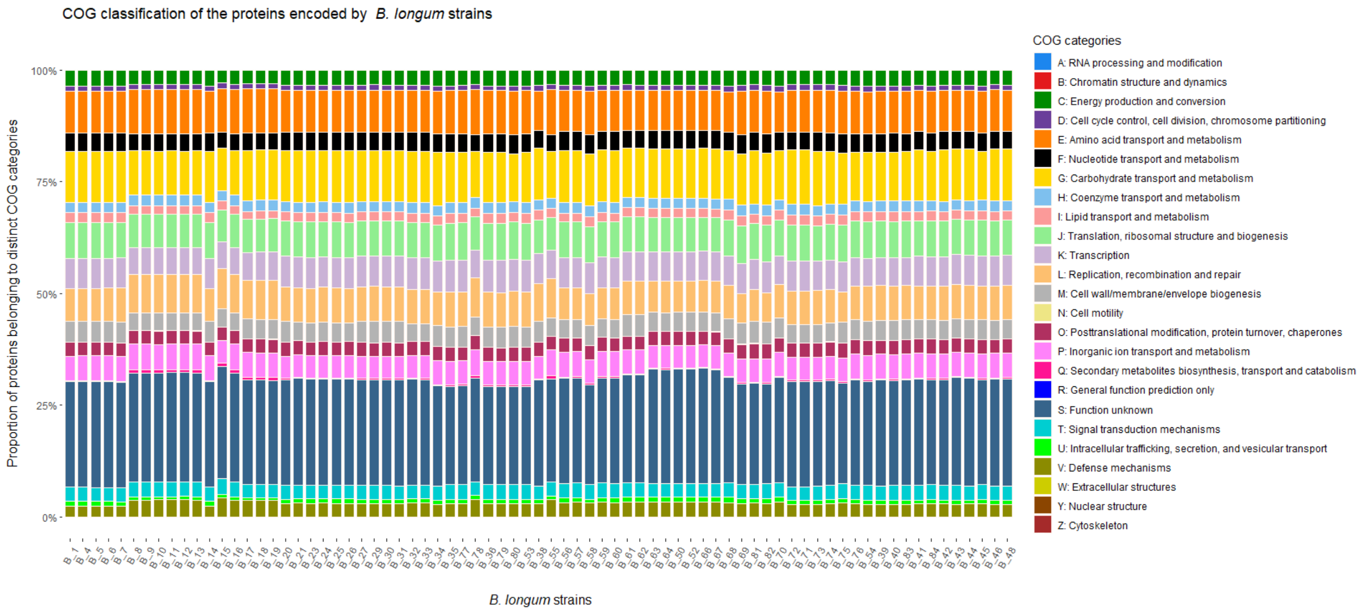

Transparent Methods

Sample collection, FISH and bacterial isolates

Infants were recruited between 2005 and 2007: five were exclusively breast-fed and four were exclusively formula-fed (**Table S2**). Faecal samples were obtained from infants at specific intervals during the first 18 months of life. For inclusion in the study, infants had to meet the following criteria: have been born at full-term (>37 weeks gestation); be of normal birth weight (>2.5 kg); be <5 weeks old and generally healthy; and be exclusively breast-fed or exclusively formula-fed [SMA Gold or SMA White (Wyeth Pharmaceuticals), to avoid supplemented formulae and to keep consistency within the formula group]. The mothers of the breast-fed infants had not consumed any antibiotics within the 3 months prior to the study and had not taken any prebiotics and/or probiotics. Ethical approval was obtained from the University of Reading Ethics Committee (Roger and McCartney, 2010). Faecal bacterial populations were assessed by FISH analysis. For details, refer to paper by Rogers and McCartney from 2010 (Roger and McCartney, 2010). Briefly, commercially synthesized 5′ Cy3-labelled oligonucleotide probes Bif164, Bac303, Chis150, ER482, Ato291, EC1531 and Lab158 (MWG Biotech) were used for detection of specific bacterial populations. 4′,6-diamidino-2-phenylindole (DAPI; 500 ng μl^{-1}) was used to enumerate the total bacterial load of samples. *Bifidobacterium* strains (n=88) were isolated from alternate faecal samples from both exclusively breast-fed (BF) and formula-fed (FF) infants. For details of the cultivation work, refer to the paper by Roger & McCartney from 2010 (Roger et al., 2010). Briefly, serially diluted aliquots of faecal homogenates (10^{-1}–10^{-8} in pre-reduced peptone water (Oxoid)) were plated in duplicate onto pre-reduced Beerens agar and incubated in an anaerobic cabinet at 37 °C for 3–5 days. Fifteen colonies were randomly selected and re-streaked on Beerens agar to purity. Pure cultures were stored on Microbank cryogenic beads (ProLab Diagnostics) at –70 °C.

The isolates were originally identified using ribosomal intergenic spacer analysis; for details, refer to the paper by Roger & McCartney (Roger and McCartney, 2010).

DNA extraction, whole-genome sequencing, assembly and annotation

Phenol-chloroform method used for genomic DNA extraction as described previously (Lawson et al., 2020). DNA isolated from pure bacterial cultures was subjected to multiplex Illumina library preparation protocol followed by sequencing on Illumina HiSeq 2500 platform (n=87) at the Wellcome Trust Sanger Institute (Hinxton, UK) or Illumina MiSeq (n=1) at Quadram Institute Bioscience (Norwich, UK) with read length of PE125 bp and PE300 bp, respectively, with an average sequencing coverage of 66.95-fold for isolates sequenced on HiSeq (minimum 46-fold, maximum 77-fold) and 231-fold for the isolate sequenced on MiSeq. Sequencing reads were checked for contamination using Kraken v1.1 (MiniKraken) (Wood and Salzberg, 2014) and pre-processed with fastp v0.20 (Chen et al., 2018) before assembling using SPAdes v3.11 with "careful" option (Bankevich et al., 2012). Contigs below 500bp were filtered out from the assemblies. Incorrectly assembled sequences were removed from further analysis (n=3). Additionally, publicly available assemblies of *Bifidobacterium* type strains (n=70) were retrieved from NCBI Genome database and all genomes were annotated with Prokka v1.13 (Seemann, 2014). The draft genomes of 75 *B. longum* isolates have been deposited to GOLD database at https://img.jgi.doe.gov, GOLD Study ID: Gs0145337.

Phylogenetic analysis

Python3 module pyANI v0.2.7 with default BLASTN+ settings was employed to calculate the average nucleotide identity (ANI) (Pritchard et al., 2016). Species delineation cut-off was set at 95% identity (Chun et al., 2018) and based on that only sequences identified as *Bifidobacterium longum* subspecies were selected for further analysis (n=75).

General feature format files of *B. longum* strains were inputted into the Roary pangenome pipeline v.3.12.0 to obtain core-genome data and the multiple sequence alignment (msa) of core genes (Mafft v7.313) (Page et al., 2015, Katoh et al., 2019). All SNP analyses of strains from individual infants was performed using Snippy v4.2.1 (Seemann, 2015) and the resulting msa was passed to the recombination removal tool Gubbins (Croucher et al., 2015). Alignments resulting from all previous steps were cleaned from poorly aligned positions using manual curation and Gblocks v0.9b where appropriate (Talavera and Castresana, 2007). The core-genome tree was generated using FastTree v2.1.9 using the GTR model with 1000 bootstrap iterations (Price et al., 2010). Snp-dists v0.2 was used to generate pairwise SNP distance matrix between strains within individual infants (Seemann et al., 2017). Altogether, the results of the SNP analysis reflected ANI results, showing that pairwise sequence identities were inversely proportional to pairwise SNP distances in *B. longum* subspecies isolates recovered from individual hosts.

Functional annotation and genome-wide association study analysis

Scoary v1.6.16 with Benjamini Hochberg correction (Brynildsrud et al., 2016) was used to associate subsets of genes with specific traits – breast-fed, formula-fed, pre-weaning, weaning and post-weaning. The p-value cut-off was set to <1e-5, sensitivity cut-off to ≥70 % and specificity cut-off to ≥90 % to report the most overrepresented genes. Functional categories (COG categories) were assigned to genes using EggNOG-mapper v0.99.3, based on the EggNOG database (bacteria) (Huerta-Cepas et al., 2017) and the abundance of genes involved in carbohydrate metabolism was calculated. As most *B. infantis* strains (12 out of 13) were isolated from breast-fed infants, we did not compare abundances of carbohydrate metabolism genes in breast-fed and formula-fed groups for this subspecies. Standalone version of dbCAN2 (v2.0.1) was used for CAZyme annotation (Zhang et

al., 2018). T-test function implemented in Microsoft Excel v16.16.20 was used to calculate statistically significant differences between average numbers of GH genes belonging to the predominant GH families ($p < 0.05$). Glycosyl hydrolase (GH) gain-loss events were predicted using Dollo parsimony implemented in Count v9.1106 (Csuros and Miklos, 2006). Snippy v4.2.1 with the "--ctgs" option, SNP-sites v2.3.3 (Page et al., 2016) and FastTree v2.1.9 (GTR model with 1000 bootstrap iterations) were used to generate the whole genome SNP tree.

Carbohydrate utilisation

To assess the carbohydrate utilisation profile, *Bifidobacterium* (1%, v/v) was grown in modified (m)MRS (pH 6.8) supplemented with cysteine HCl at 0.05% and 2% (w/v) of selected carbohydrates (HMOs obtained from Glycom, Hørsholm, Denmark) as described previously (Lawson et al., 2020), except for pectin and mucin which were added at 1% (w/v). Growth was determined over a 48-h period using Tecan Infinite 50 (Tecan Ltd, UK) microplate spectrophotometer at OD_{595}. Experiments were performed in biologically independent triplicates, and the plate reader measurements were taken automatically every 15 min following 60 s of shaking at normal speed. Due to the expected drop in initial OD values (i.e. recorded between T_0 and T_1) growth data were expressed as mean of the replicates between T_2 (30 min) and T_{end} (48-h).

High-performance anion-exchange chromatography (HPAEC)

Mono-, di- and oligo- saccharides present in the spent media samples were analyzed on a Dionex ICS-5000 HPAEC system operated by the Chromeleon software version 7 (Dionex, Thermo Scientific). Samples were bound to a Dionex CarboPac PA1 (Thermo Scientific) analytical column (2 × 250 mm) in combination with a CarboPac PA1 guard column (2 × 50 mm), equilibrated with 0.1 M NaOH. Carbohydrates were detected by pulsed amperometric detection (PAD). The

system was run at a flow rate of 0.25 mL/min. The separation was done using a stepwise gradient going from 0.1 M NaOH to 0.1 M NaOH–0.1 M sodium acetate (NaOAc) over 10 min, 0.1 M NaOH–0.3 M NaOAc over 25 min followed by a 5 min exponential gradient to 1 M NaOAc, before reconditioning with 0.1 M NaOH for 10 min. Commercial glucose, cellobiose, fucose, lactose and lacto-*N*-neotetraose (LNnT) were used as external standards.

Proteomics

B. longum subsp. *longum* strain 25 (B_25) was grown in triplicate in mMRS supplemented with cysteine HCl at 0.05% and 2% (w/v) glucose, cellobiose or LNnT as a sole carbon source. *B. longum* subsp. *longum* strain 71 (B_71) was grown in triplicate in mMRS supplemented with cysteine HCl at 0.05% and either 2% (w/v) glucose or 2'-fucosyllactose (2'-FL) as a sole carbon source. Cell pellets from 50 mL samples (at the mid-exponential growth phase) were collected by centrifugation (4500 × g, 10 min, 4 °C) and washed three times with PBS pH 7.4. Cells were resuspended in 50 mM Tris-HCl pH 8.4 and disrupted by bead-beating in three 60 s cycles using a FastPrep24 (MP Biomedicals, CA). Protein concentration was determined using a Bradford protein assay (Bio-Rad, Germany). Protein samples, containing 50 µg total protein, were separated by SDS-PAGE with a 10% Mini-PROTEAN gel (Bio-Rad Laboratories, CA) and then stained with Coomassie brilliant blue R250. The gel was cut into five slices, after which proteins were reduced, alkylated, and in-gel digested as previously described (Arntzen et al., 2015). Peptides were dissolved in 2% acetonitrile containing 0.1% trifluoroacetic acid and desalted using C18 ZipTips (Merck Millipore, Germany). Each sample was independently analysed on a Q-Exactive hybrid quadrupole-orbitrap mass spectrometer (Thermo Scientific) equipped with a nano-electrospray ion source. MS and MS/MS data were acquired using Xcalibur (v.2.2 SP1). Spectra were analysed using MaxQuant 1.6.1.0 (Cox and Mann, 2008) and searched against a sample-

specific database generated from the B_25 and B_71 genomes. Proteins were quantified using the MaxLFQ algorithm (Cox et al., 2014). The enzyme specificity was set to consider tryptic peptides and two missed cleavages were allowed. Oxidation of methionine, N-terminal acetylation and deamidation of asparagine and glutamine and formation of pyro-glutamic acid at N-terminal glutamines were used as variable modifications, whereas carbamidomethylation of cysteine residues was used as a fixed modification. All identifications were filtered in order to achieve a protein false discovery rate (FDR) of 1% using the target-decoy strategy. A protein was considered confidently identified if it was detected in at least two of the three biological replicates in at least one glycan substrate. The MaxQuant output was further explored in Perseus v.1.6.1.1 (Tyanova et al., 2016). The proteomics data have been deposited to the ProteomeXchange Consortium (http://proteomecentral.proteomexchange.org) via the PRIDE partner repository with dataset identifier PXD017277.

Supplemental References

ARNTZEN, M. O., KARLSKAS, I. L., SKAUGEN, M., EIJSINK, V. G. H. & MATHIESEN, G. 2015. Proteomic Investigation of the Response of Enterococcus faecalis V583 when Cultivated in Urine. *Plos One,* 10.

BANKEVICH, A., NURK, S., ANTIPOV, D., GUREVICH, A. A., DVORKIN, M., KULIKOV, A. S., LESIN, V. M., NIKOLENKO, S. I., PHAM, S., PRJIBELSKI, A. D., PYSHKIN, A. V., SIROTKIN, A. V., VYAHHI, N., TESLER, G., ALEKSEYEV, M. A. & PEVZNER, P. A. 2012. SPAdes: a new genome assembly algorithm and its applications to single-cell sequencing. *J Comput Biol,* 19**,** 455-77.

BRYNILDSRUD, O., BOHLIN, J., SCHEFFER, L. & ELDHOLM, V. 2016. Rapid scoring of genes in microbial pan-genome-wide association studies with Scoary. *Genome Biol,* 17**,** 238.

CHEN, S., ZHOU, Y., CHEN, Y. & GU, J. 2018. fastp: an ultra-fast all-in-one FASTQ preprocessor. *Bioinformatics,* 34**,** i884-i890.

CHUN, J., OREN, A., VENTOSA, A., CHRISTENSEN, H., ARAHAL, D. R., DA COSTA, M. S., ROONEY, A. P., YI, H., XU, X. W., DE MEYER, S. & TRUJILLO, M. E. 2018. Proposed minimal standards for the use of genome data for the taxonomy of prokaryotes. *International Journal of Systematic and Evolutionary Microbiology,* 68**,** 461-466.

COX, J., HEIN, M. Y., LUBER, C. A., PARON, I., NAGARAJ, N. & MANN, M. 2014. Accurate Proteome-wide Label-free Quantification by Delayed Normalization and Maximal

Peptide Ratio Extraction, Termed MaxLFQ. *Molecular & Cellular Proteomics,* 13, 2513-2526.
COX, J. & MANN, M. 2008. MaxQuant enables high peptide identification rates, individualized p.p.b.-range mass accuracies and proteome-wide protein quantification. *Nature Biotechnology,* 26, 1367-1372.
CROUCHER, N. J., PAGE, A. J., CONNOR, T. R., DELANEY, A. J., KEANE, J. A., BENTLEY, S. D., PARKHILL, J. & HARRIS, S. R. 2015. Rapid phylogenetic analysis of large samples of recombinant bacterial whole genome sequences using Gubbins. *Nucleic Acids Research,* 43.
CSUROS, M. & MIKLOS, I. 2006. A probabilistic model for gene content evolution with duplication, loss, and horizontal transfer. *Research in Computational Molecular Biology, Proceedings,* 3909, 206-220.
HUERTA-CEPAS, J., FORSLUND, K., COELHO, L. P., SZKLARCZYK, D., JENSEN, L. J., VON MERING, C. & BORK, P. 2017. Fast Genome-Wide Functional Annotation through Orthology Assignment by eggNOG-Mapper. *Molecular Biology and Evolution,* 34, 2115-2122.
KATOH, K., ROZEWICKI, J. & YAMADA, K. D. 2019. MAFFT online service: multiple sequence alignment, interactive sequence choice and visualization. *Brief Bioinform,* 20, 1160-1166.
LAWSON, M. A. E., O'NEILL, I. J., KUJAWSKA, M., GOWRINADH JAVVADI, S., WIJEYESEKERA, A., FLEGG, Z., CHALKLEN, L. & HALL, L. J. 2020. Breast milk-derived human milk oligosaccharides promote Bifidobacterium interactions within a single ecosystem. *ISME J,* 14, 635-648.
PAGE, A. J., CUMMINS, C. A., HUNT, M., WONG, V. K., REUTER, S., HOLDEN, M. T. G., FOOKES, M., FALUSH, D., KEANE, J. A. & PARKHILL, J. 2015. Roary: rapid large-scale prokaryote pan genome analysis. *Bioinformatics,* 31, 3691-3693.
PAGE, A. J., TAYLOR, B., DELANEY, A. J., SOARES, J., SEEMANN, T., KEANE, J. A. & HARRIS, S. R. 2016. SNP-sites: rapid efficient extraction of SNPs from multi-FASTA alignments. *Microbial Genomics,* 2.
PRICE, M. N., DEHAL, P. S. & ARKIN, A. P. 2010. FastTree 2-Approximately Maximum-Likelihood Trees for Large Alignments. *Plos One,* 5.
PRITCHARD, L., GLOVER, R. H., HUMPHRIS, S., ELPHINSTONE, J. G. & TOTH, I. K. 2016. Genomics and taxonomy in diagnostics for food security: soft-rotting enterobacterial plant pathogens. *Analytical Methods,* 8, 12-24.
ROGER, L. C., COSTABILE, A., HOLLAND, D. T., HOYLES, L. & MCCARTNEY, A. L. 2010. Examination of faecal Bifidobacterium populations in breast- and formula-fed infants during the first 18 months of life. *Microbiology-Sgm,* 156, 3329-3341.
ROGER, L. C. & MCCARTNEY, A. L. 2010. Longitudinal investigation of the faecal microbiota of healthy full-term infants using fluorescence in situ hybridization and denaturing gradient gel electrophoresis. *Microbiology-Sgm,* 156, 3317-3328.
SEEMANN, T. 2014. Prokka: rapid prokaryotic genome annotation. *Bioinformatics,* 30, 2068-9.
SEEMANN, T. 2015. snippy: fast bacterial variant calling from NGS reads. Available at: https://github.com/tseemann/snippy.
SEEMANN, T., PAGE, A. J. & KLOTZL, F. 2017. snp-dists: Pairwise SNP distance matrix from a FASTA sequence alignment. Available at: https://github.com/tseemann/snp-dists.

TALAVERA, G. & CASTRESANA, J. 2007. Improvement of phylogenies after removing divergent and ambiguously aligned blocks from protein sequence alignments. *Systematic Biology,* 56**,** 564-577.

TYANOVA, S., TEMU, T., SINITCYN, P., CARLSON, A., HEIN, M. Y., GEIGER, T., MANN, M. & COX, J. 2016. The Perseus computational platform for comprehensive analysis of (prote)omics data. *Nature Methods,* 13**,** 731-740.

WOOD, D. E. & SALZBERG, S. L. 2014. Kraken: ultrafast metagenomic sequence classification using exact alignments. *Genome Biology,* 15.

ZHANG, H., YOHE, T., HUANG, L., ENTWISTLE, S., WU, P., YANG, Z., BUSK, P. K., XU, Y. & YIN, Y. 2018. dbCAN2: a meta server for automated carbohydrate-active enzyme annotation. *Nucleic Acids Res,* 46**,** W95-W101.
